# The maths of memory

**DOI:** 10.7554/eLife.26754

**Published:** 2017-04-28

**Authors:** Jose Borghans, Ruy M Ribeiro

**Affiliations:** 1 Laboratory of Translational Immunology, University Medical Center Utrecht, Utrecht, The NetherlandsJ.Borghans@umcutrecht.nl; 2 Laboratório de Biomatemática, Faculdade de Medicina, Universidade de Lisboa, Lisbon, Portugal

**Keywords:** T cell homeostasis, BrdU labelling, Ki67, memory T cells, mathematical modelling, Mouse

## Abstract

Mathematical modeling reveals that long-term immunological memory is maintained in a manner that is even more dynamic than previously thought.

**Related research article** Gossel G, Hogan T, Cownden D, Seddon B, Yates AJ. 2017. Memory CD4 T cell subsets are kinetically heterogeneous and replenished from naive T cells at high levels. *eLife* **6**:e23013. doi: 10.7554/eLife.23013

Immunological memory – the ability of the body to ‘remember’ and fight previously encountered pathogens – forms the basis of vaccination, which is one of the most important discoveries in the history of medicine. However, despite the enormous success of vaccination, we still do not fully understand how the body maintains long-term immunological memory, and this gap in our knowledge is hindering attempts to develop ‘next-generation vaccines’ and efforts to deploy immune cells called T cells against cancer.

The immune system contains many different types of cells. Some of these cells can recognize pathogens without ever having encountered them. However, other immune cells – including T cells – ‘learn on the job’: on first encountering a pathogen, these cells respond relatively slowly, but later form a 'memory' to respond more efficiently. To better understand how long-term immunological memory is maintained, we need to learn more about the dynamics of memory T cells. In particular, it will be necessary to understand the relative contribution of the following processes: the recruitment of new T cells (which are known as naive T cells) into the pool of memory T cells; the renewal of memory T cells by cell division; and the survival of individual memory T cells. However, quantifying these processes is challenging.

Previous research using cancer patient data indicated that memory T cells have a relatively short lifespan (Michie et al., 1992). Then, almost 20 years ago, DNA-labeling techniques led to a breakthrough in the study of T-cell dynamics by allowing researchers to track how rapidly T cells divide and die (Hellerstein et al., 1999). These methods were later used to confirm that memory T cells live for six months or less in healthy humans (Westera et al., 2013), whereas naive T cells can live for up to nine years (Vrisekoop et al., 2008). Thus, a long life is not a key characteristic of memory T cells. Instead, immunological memory, which can last for a lifetime (Crotty and Ahmed, 2004), is maintained by relatively short-lived cells. However, it remains unclear to what extent the pool of memory T cells is maintained by the division of existing memory T cells or through the recruitment of naive T cells to the pool. The latter process is thought to play only a minor role because naive T cells are highly variable, and the chance of a given naive T cell entering the pool of memory T cells is thus extremely low.

Now, in eLife, immunologists from the University of Glasgow, the Icahn School of Medicine at Mount Sinai and the Royal Free Hospital – Graeme Gossel, Thea Hogan, Daniel Cownden, Benedict Seddon and Andrew Yates – report how they have used two independent approaches to study how T-cell memory is maintained (Gossel et al., 2017). In a technique called ‘temporal fate mapping’, Gossel et al. used the cancer drug busulfan to kill hematopoietic stem cells (that is, stem cells that go on to become blood cells like T cells) in the bone marrow of mice, while leaving their peripheral T-cell pools intact. They then transplanted bone marrow cells from donor mice, which mature into T cells that differ only in one protein marker (CD45) expressed on their surface. Thus, in these mice, the researchers were able to distinguish newly formed T cells from the original T cells, and to track which cells were being replaced and measure their dynamics (Figure 1A).

**Figure 1.**
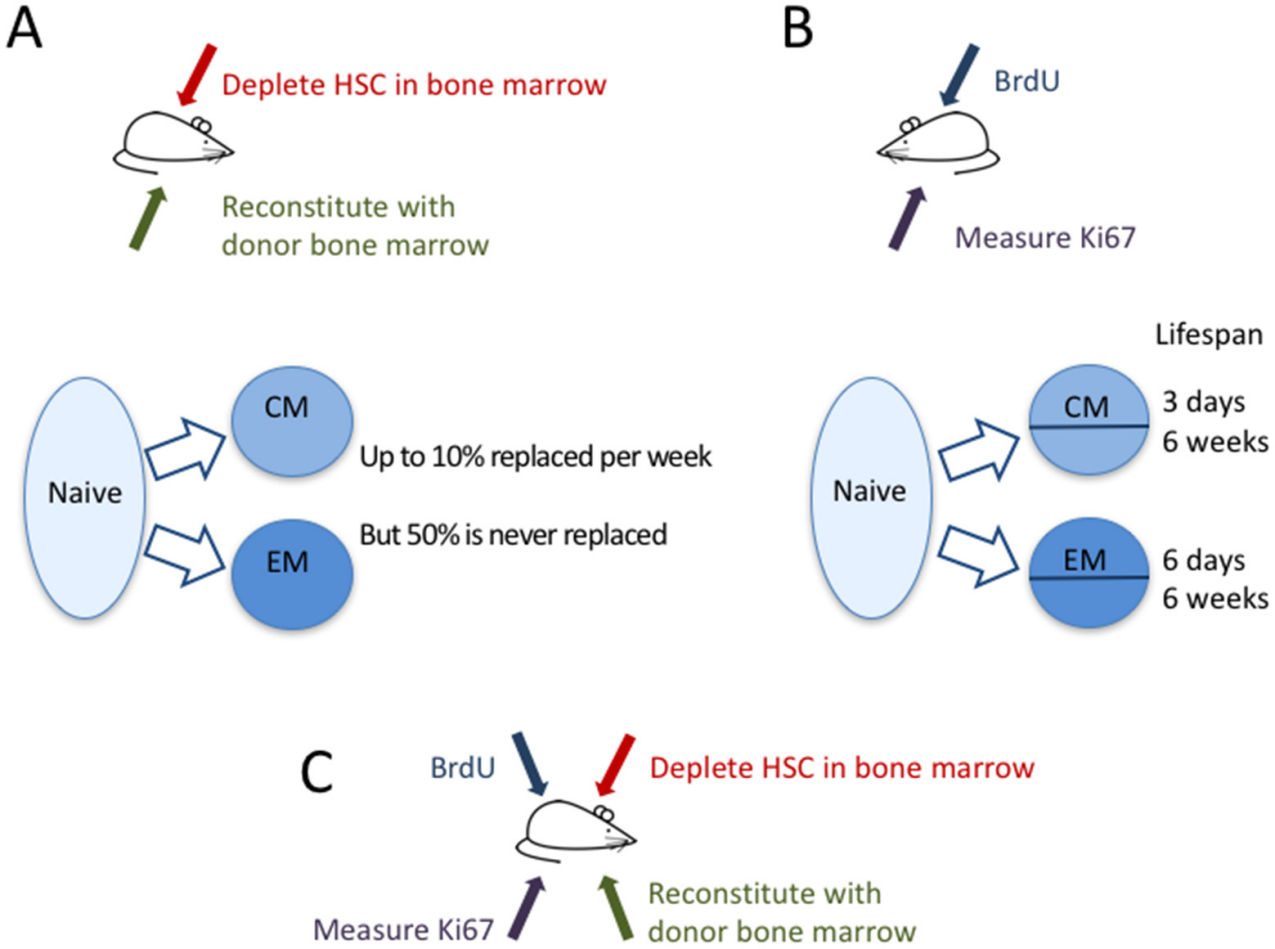
The dynamics of memory T cells. Gossel et al. used two experimental approaches to study the dynamics of memory T cells in mice. (**A**) To quantify the influx of naive T cells into the pool of existing memory T cells, they used the drug busulfan to selectively kill hematopoietic stem cells (HSC) in the bone marrow of CD45.1+ mice (red arrow), and then added new cells derived from bone marrow of CD45.2+ mice (green arrow). Naive T cells (light blue bubble) replaced around 10% of central memory T cells (CM) and about 6% of effector memory T cells (EM) per week (blue arrows). About half of the resident memory T-cell population was never replaced. (**B**) In a second experiment, the DNA of memory T cells was labeled with the marker BrdU to measure how rapidly cells in different memory T-cell subpopulations divide and die. By measuring the marker Ki67 (purple arrow), they could classify CM and EM cells into recently divided (Ki67+) and not recently divided (Ki67-) cells. Both the CM and the EM pools contained cells with fast and slow dynamics: the short-lived cells lived around three days and six days, respectively, while the long-lived cells lived for around six weeks in both subpopulations. (**C**) In the future, it might be useful to combine these two approaches to compare the cell division and death of recently recruited (CD45.2+) and pre-existing memory T cells (CD45.1+).

Even though the mice did not have any infections to activate naive T cells, there appeared to be a constant large influx of naive T cells into the pool of memory T cells. The memory T-cell pool has different subpopulations, including central memory T cells and effector memory T cells, which can be distinguished by the different protein markers found on their surface. Gossel et al. discovered that naive T cells replaced around 10% of the central memory T cells every week. For effector memory T cells this figure was around 6% per week in young adults and 1% per week in older mice. Importantly, they showed that by neglecting this large flux from the naive T-cell pool into the memory pool, previous studies may have significantly overestimated the lifespan of memory T cells.

Despite this rapid replacement of memory T cells by new naive T cells, around 50% of the pool of memory T cells that had formed before the mice were eight weeks old was never replaced by new naive T cells. Thus, although a substantial fraction of the memory T-cell pool is replaced at high rates throughout life, an equally large proportion is kept from an early age. The question still remains whether these ‘original’ T cells simply have a long lifespan or are maintained through cell division.

To better understand the dynamics of the different T-cell sub-populations, Gossel et al. labeled the DNA of cells undergoing cell division and measured the protein Ki67, which cells naturally express during division (Figure 1B). The Ki67 marker was then used as a ‘time stamp’ that tagged cells that had recently divided, while DNA-labeling was used to trace the dynamics of both ‘stamped’ and ‘non-stamped’ cells. Gossel et al. show for the first time that both the central memory and the effector memory T-cell pool are composed of T-cell subsets with intrinsically different dynamics. In both pools around half of the cells live for just a few days while the other half, on average, live for around six weeks.

This study beautifully combines detailed quantitative experiments with mathematical models, and thereby reveals important insights into the long-term maintenance of memory T cells. However, the work also raises many questions. For example, it has recently been shown that the pool of memory T cells of laboratory mice resembles that of human babies more than that of human adults (Beura et al., 2016). If new naive T cells replace up to 10% of the memory T-cell pool per week even in clean laboratory environments, one may wonder how much they would replace in humans, who are continuously exposed to pathogens.

It is also still not clear what is driving these cells into the memory T-cell pool and if chronic latent infections continuously recruit new naive T cells into the memory pool. Nevertheless, a substantial fraction of the memory T-cell pool appears to be resistant and cannot be replaced by new cells from the naive T-cell pool. Future research should address what makes these T cells resistant and whether they cannot be replaced even during infections. Last but not least, one may wonder, what is the benefit of combining a pool of memory T cells that is never replaced with one that is quickly and continuously replaced?

## References

[bib1] Beura LK, Hamilton SE, Bi K, Schenkel JM, Odumade OA, Casey KA, Thompson EA, Fraser KA, Rosato PC, Filali-Mouhim A, Sekaly RP, Jenkins MK, Vezys V, Haining WN, Jameson SC, Masopust D (2016). Normalizing the environment recapitulates adult human immune traits in laboratory mice. Nature.

[bib2] Crotty S, Ahmed R (2004). Immunological memory in humans. Seminars in Immunology.

[bib3] Gossel G, Hogan T, Cownden D, Seddon B, Yates AJ (2017). Memory CD4 T cell subsets are kinetically heterogeneous and replenished from naive T cells at high levels. eLife.

[bib4] Hellerstein M, Hanley MB, Cesar D, Siler S, Papageorgopoulos C, Wieder E, Schmidt D, Hoh R, Neese R, Macallan D, Deeks S, McCune JM (1999). Directly measured kinetics of circulating T lymphocytes in normal and HIV-1-infected humans. Nature Medicine.

[bib5] Michie CA, McLean A, Alcock C, Beverley PC (1992). Lifespan of human lymphocyte subsets defined by CD45 isoforms. Nature.

[bib6] Vrisekoop N, den Braber I, de Boer AB, Ruiter AF, Ackermans MT, van der Crabben SN, Schrijver EH, Spierenburg G, Sauerwein HP, Hazenberg MD, de Boer RJ, Miedema F, Borghans JA, Tesselaar K (2008). Sparse production but preferential incorporation of recently produced naive T cells in the human peripheral pool. PNAS.

[bib7] Westera L, Drylewicz J, den Braber I, Mugwagwa T, van der Maas I, Kwast L, Volman T, van de Weg-Schrijver EH, Bartha I, Spierenburg G, Gaiser K, Ackermans MT, Asquith B, de Boer RJ, Tesselaar K, Borghans JA (2013). Closing the gap between T-cell life span estimates from stable isotope-labeling studies in mice and humans. Blood.

